# Oncogenic ACSM6 impairs CD8^+^ T cell-based immune response in bladder cancer

**DOI:** 10.1186/s40364-024-00657-y

**Published:** 2024-09-27

**Authors:** Zhenyu Nie, Bolong Liu, Jinhui Liu, Xiongbing Zu, Juanhua Wang, Jinbo Chen, Benyi Fan, Dingshan Deng

**Affiliations:** 1 Department of Urology, Xiangya Hospital, Central South University, Changsha, China; 2 National Clinical Research Center for Geriatric Disorders, Xiangya Hospital, Central South University, Changsha, China; 3 Department of andrology, The First Affiliated Hospital Of University Of South China, University Of South China, Hengyang, China; 4 Department of Urology, Hunan Provincial People’s Hospital, Changsha, China; 5 Department of Urology, The First Affiliated Hospital of Hunan Normal University, Hunan Normal University, Changsha, China; 6 Hunan Vocational and Technical College of Environmental Biology, Changsha, China

**Keywords:** Bladder cancer, Tumor microenvironment, Immunotherapy resistance, Non-inflammatory immune microenvironment, ACSM6

## Abstract

Resistance to immunotherapy in bladder cancer has greatly limited its clinical application. Through single-cell sequencing, we determined that ACSM6, an oncogene that is highly expressed in bladder cancer, promotes the abilities of proliferation, cloning, migration, and invasion. The key point is that ACSM6 can also lead to a non-inflammatory immune microenvironment by inhibiting the chemotaxis and tumor killing ability of CD8^+^ T cells. Survival analysis revealed that high ACSM6 expression was associated with shorter overall survival in the immunotherapy cohort. In summary, ACSM6 is expected to become a novel biomarker for predict bladder cancer progression.

**Supplementary Information:**

The online version contains supplementary material available at 10.1186/s40364-024-00657-y.

## To the editor

Until recent immunotherapy breakthroughs, bladder urothelial carcinoma (BLCA), the most common urinary tumor, had seen limited progress in chemotherapy-based treatment [1]. Immune checkpoint inhibitors (ICIs) targeting PD-1/PD-L1 have improved outcomes, especially in platinum-resistant BLCA. FDA-approved ICIs show a 13-24% response [2], but immunotherapy resistance limits their wider use [3].

Acyl-CoA synthase family members (ACSMs) catalyze fatty acid activation, initiating fatty acid metabolism. Members of this familiy facilitate medium-chain fatty acid synthesis and metabolism. In addition, ACSMs modulate tumor development via fatty acid metabolism. High ACSM3 expression promotes hepatocellular carcinoma cell invasion, metastasis, and poor prognosis via fatty acid oxidation [4]. ACSM1/3 regulates androgen receptor signaling in prostate cancer by inhibiting ferroptosis and tumor progression [5]. ACSM4 has an unclear but adverse role in the prognosis of triple-negative breast cancer [6]. Notably, ACSM6 is understudied. Our team discovered ACSM6-mediated immunotherapy resistance in BLCA [7]. We found that ACSM6 overexpression in BLCA predicts a non-inflammatory tumor immune microenvironment (TIME) and immunotherapy responses [8, 9]. This study deepens understanding of ACSM6 role in the TIME of BLCA.

**Fig. 1:**
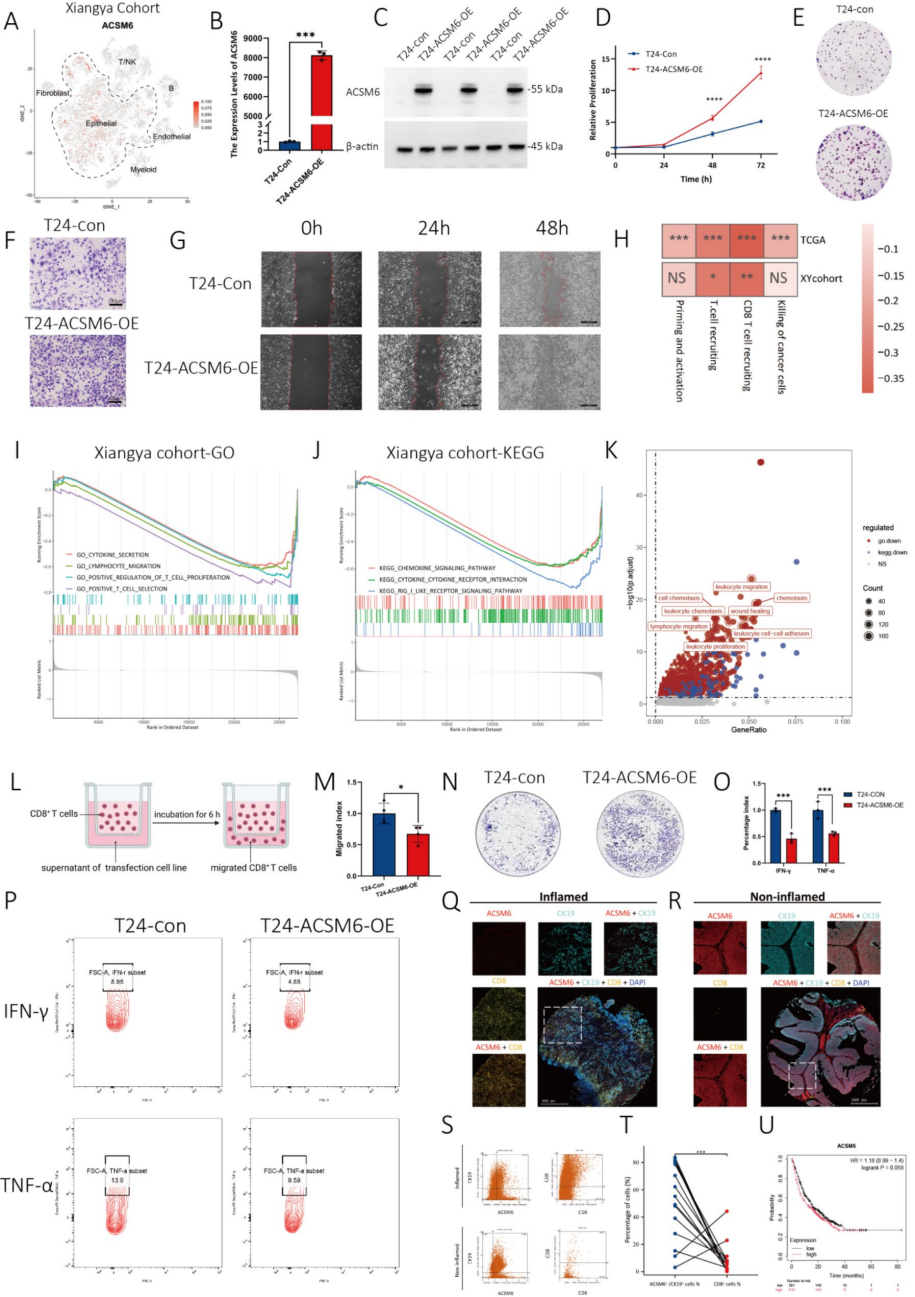
ACSM6 mediates inhibitory tumor immune microenvironment in bladder cancer and leads to drug resistance in immunotherapy. **(A)** Xiangya Single cell cohort to illuminate ACSM6 high expression cells. Over-expression fold change has been verified with RT-qPCR **(B)** and Western blotting **(C)**. **(D)**. Cell proliferation assay. **(E)** Colony formation assay. **(F)** Transwell assay. **(G)** Wound healing assay. **(H)** Simple heat map to illuminate the impact of high expression of ACSM6 on immune function in TCGA and Xiangya cohorts. GSEA analysis of GO **(I)** and KEGG **(J)** in Xiangya cohort with high expression of ACSM6. **(K)** GO and KEGG pathways down-regulated after over-expression of ACSM6. **(L)** Schematic map of chemotaxis assay. **(M)** Cells differential fold change of chemotaxis assay. **(N)** Co-cultivation killing assay. **(O)** Over-expression of ACSM6 inhibits the fold change of TNF-α and IFN-γ in CD8^+^ T cells. **(P)** Flow cytometry analysis of the expression level of TNF-α and IFN-γ in CD8^+^ T cells. Multi-color immuno-fluorescence of inflamed **(Q)** and non-inflamed **(R)** tumor tissue. **(S)** Flow cytometry like analysis describes the co-localization relationship between ACSM6 and CD8^+^ T cells and it’s quantitative analysis **(T)**. Pairing statistics of ACSM6^+^/CK19^+^ cells and CD8^+^T cells **(U)**. Kaplan-Meier plotter interpret the high expression of ACSM6 shorten the OS of immunotherapy

Analysis of the Xiangya and PRJNA662018 cohorts [10] indicated ACSM6 specificity for epithelial cells, excluding stromal or immune cells (Fig. 1A and Supplementary Fig. 1). We overexpressed ACSM6 in bladder cancer T24 cells via lentiviral transfection, as confirmed by RT-qPCR and western blotting (Fig. 1B-C, Supplementary Fig. 2). Subsequent studies evaluated the effect of ACSM6 on bladder cancer cell proliferation, invasion, and migration (Fig. 1D and G, and Supplementary Figs. 3–5). These findings demonstrate that enhanced ACSM6 expression promotes malignant behaviors. Previous studies have indicated that ACSM6 overexpression in the BLCA leads to a non-inflammatory TIME and ICIs failure. TCGA and Xiangya cohort analyses confirmed a negative correlation between ACSM6 and antitumor immunity markers (Fig. 1H). GO and KEGG analyses of Xiangya and PRJNA662018 show downregulation of immune-related pathways, such as cytokine secretion; lymphocyte migration; T cell activation, chemotaxis, and migration; and cytokine receptor interaction, in high ACSM6-BLCA (Fig. 1I-J and Supplementary Figs. 6–7). Transcriptome sequencing of T24-ACSM6-OE cells validated these findings, revealing downregulation of leukocyte migration, chemotaxis, and proliferation pathways (Fig. 1K). Chemotaxis (diagrammed as Fig. 1L) and tumor-killing assays demonstrate that ACSM6 inhibits the CD8^+^ T cell chemotaxis and killing abilities (Fig. 1M and N, and Supplementary Fig. 8), while flow cytometry shows reduced TNF-α and IFN-γ of CD8^+^ T cells in ACSM6-OE co-cultures (Fig. 1O-P). These results suggest that ACSM6 impairs CD8^+^ T cell-mediated antitumor immunity in BLCA. We validated the ACSM6-TIME correlation in BLCA immunotherapy tissue microarrays using immunofluorescence staining. ACSM6^+^ and CK19^+^ cells colocalized but segregated from CD8^+^ cells (Fig. 1Q-R). Flow-cytometry like revealed a higher number of ACSM6^+^ CK19^+^ cells in non-inflammatory tumors than in inflammatory cells, inversely correlating with CD8^+^ T cells. Further, multi-color staining confirmed that ACSM6 was exclusive to CK19^+^ tumor cells, not PDGFR-α^+^ fibroblasts, indicating ACSM6 specificity to tumor cells (Supplementary Fig. 9). In an extensive immunotherapy dataset encompassing diverse tumor types [11], although statistical significance was not definitively reached, a notable trend emerged, suggesting a correlation between elevated ACSM6 expression and reduced overall survival among patients undergoing immunotherapy.

This study confirms and expands prior findings by identifying ACSM6 as an oncogene in bladder cancer that promotes proliferation and metastasis and inhibits CD8^+^ T-cell function. High ACSM6 levels inhibits T cell infiltration and foster a non-inflammatory tumor microenvironment. Despite its similar expression levels in BLCA and normal tissues [7], ACSM6 may serve as a biomarker and therapeutic target for tumor microenvironment assessment. However, its absence in non-primate genomes hinders in vivo functional studies. In general, ACSM6 is a novel credible bladder cancer biomarker for predicting tumor progression and immunotherapy response.

## Electronic supplementary material

Below is the link to the electronic supplementary material.

Supplementary Material 1

Supplementary Material 2

## Data Availability

The data underlying this article are available in the article. Request for additional information can be made to the corresponding author.
