# Supplementary material for: Oncogenic ACSM6 impairs CD8+ T cell-based immune response in bladder cancer
Source: Biomark Res. 2024 Sep 27;12:112. doi: 10.1186/s40364-024-00657-y (PMC11437728; doi:10.1186/s40364-024-00657-y)
Supplement: Supplementary file 1 — Supplementary Material 1 [file 40364_2024_657_MOESM1_ESM.docx]

**Materials and Methods**

**Xiangya cohort**

We collected tissue samples from 58 bladder cancer patients meeting the criteria at Xiangya Hospital, along with 13 normal tissues adjacent to the tumors for RNA sequencing (RNA-seq), named the Xiangya Bladder Cancer Cohort (XY-BLCA cohort). Additionally, we performed single-cell RNA sequencing on three muscle-invasive bladder cancer samples, which we named the Xiangya ScRNA Cohort (XY-scRNA cohort). We assembled a cohort of 58 patients diagnosed with muscle-invasive bladder cancer who received immune therapy, undergoing Transurethral Resection of Bladder Tumor (TURBT) or Radical Cystectomy (RC) after at least two cycles of neoadjuvant immunotherapy, named the Xiangya Bladder Cancer Immunotherapy Cohort (XY-immunotherapy cohort). Furthermore, we constructed a tissue microarray (TMA) containing 50 BLCA samples and 28 paired normal tissue samples from the vicinity, named the Xiangya TMA Cohort (XY-TMA cohort).

**Public database**

The Cancer Genome Atlas (TCGA) database was obtained from the UCSC Xena data portal (https://xenabrowser.net/). Public single-cell RNA sequencing (scRNA-seq) data consisting of 8 BLCA and 3 normal tissue samples (PRJNA662018, Z. Chen, L. Zhou, L. Liu, Y. Hou, M. Xiong, Y. Yang, J. Hu, K. Chen, Nat. Commun. 2020, 11, 5077.) were download.

**Cell lines and cell culture**

The human bladder cancer cell lines T24, 5637, J82 and UMUC-3 were purchased from the American Type Culture Collection (ATCC, Manassas, VA, USA). All cells were cultured in an incubator containing 5% CO_2_ and 95% air at 37℃. All cell culture media and FBS were obtained from Gibco (Grand Island, NY, USA). J82 and UMUC-3 cells were cultured in DMEM (10% FBS), T24 and 5637 cells were cultured in RPMI 1640 (10% FBS), and SV-HUC-1 cells were cultured in F-12 K (10% FBS) medium.

**Stable cell line construction**

The packaging of ACSM6 overexpressed virus was provided by oebiotech Company (Shanghai, China), with the following primers: ACSM6 forward:5'- CCAACTTTGTGCCAACCGGTCGCCACCATGCTAGGCCGATTTCAACC-3' and reverse:5'-GTCAATGCCAACTCTGAGCTTCAATCTCTGACCCAATGCATTGGCAAC-3'.The lentivirus vector plasmid GV341(Ubi-MCS-3FLAG-SV40-puromycin). Cells were seeded in a six-well plate at a density of 0.5×10^5^ cells/ml using complete culture medium, and cultured under 37℃ 5% CO_2_ conditions for 16h. When the cell density reached 50%, according to the manufacturer’s protocol, the virus was transfected into the cells. 2 ug/ml of purromycin (Amersco, USA) was added to the complete culture medium for continuous selection for 5 days. Subsequently, validation was performed using qRT-PCR and Western blot.

**qRT-PCR**

Total RNA was extracted from stable cell lines using the RaPure Total RNA Kit (Magentec, Canton, China). Reverse transcription to cDNA was performed using HiScript®III All-in-one RT SuperMix Perfect for qPCR (Vazyme, R333). cDNA was amplified with an Applied Biosystems QuantStudio 5 Real-Time PCR instrument (Thermo Fisher Scientific, Waltham, MA, USA) using ChamQ Universal SYBR qPCR Master Mix (Vazyme, Q711). Relative expression levels of target mRNA were calculated with ACTB as the internal control. The primers were designed and synthesized by Sangon Biotech (Shanghai, China). The 2^−∆∆Ct^ comparative method was applied to calculate the relative ACSM6 mRNA expression.

**Western blot**

After PBS washing, cells were lysed using RIPA buffer containing 1% Protease and Phosphatase Inhibitor Cocktail and 5×SDS-PAGE loading Buffer (NCM biotech, China). Protein concentration was determined by the BCA protein assay kit (NCM biotech, China). The protein samples were loaded onto an electrophoresis gel with loading buffer at both ends, followed by electrophoresis, blotting, blocking, incubation with primary antibodies, and further incubation with secondary antibodies. Imaging was performed using an Bio-Rad imaging system. Primary antibodies include: Anti-ACSM6 antibody (PA5-60768, Invitrogen, Carlsbad, USA) and Anti-Beta Actin antibody (66009-1-Ig, Proteintech, Deansgate, USA). The second antibody was HRP goat anti-rabbit (ab6721, Abcam, Combridge, USA).

**Cell Counting Kit-8 for proliferation**

The bladder cancer cell line digested by trypsin was seeded at a concentration of 1×10^4^/ml, 100ul/well in a 96-well plate, and cultured in complete medium. 100ul/well of PBS was added peripherally for enclosure. After 4-5 hours, when the tumor cells completely adhered to the wall, the corresponding wells were removed of medium at 0h, 24h, 48h, 72h, and 96h, and 100ul of 10% Cell Counting Kit-8 (CK04, Dojindo Laboratories, Tokyo, Japan) reagent was added. After 1.5 hours of incubation, the absorbance was measured using a spectrophotometer.

**Wound healing experiment**

Using a marker pen to evenly mark lines on the back of a six-well plate, then inoculate the cells at a density of 5×10^5^ cells/well, and add medium for cultivation. When the cells adhere to the wall and the growth density reaches 80%, replace the medium in the six-well plate with fresh serum-free medium, use a 100ul pipette tip to evenly and vertically scratch the wells after 6 hours, wash three times with PBS, take pictures under a microscope, then place the six-well plate in a culture environment at 37°C, 5% CO_2_ for another 24 hours, wash three times with PBS, take pictures under a microscope again and analyze.

**Colony formation and proliferation assay**

A total of 500 T24 cells cells were resuspended in 0.2 mL of medium, evenly added to a 6-well plate containing 1.8 mL of 10% FBS medium, and cultured in the incubator for 2 weeks. After 2 weeks, the 6-well plate was taken out, and the cells were observed using a microscope to grow in a single colony. The culture medium was discarded, the PBS was gently washed three times, and the cells were dyed with 0.5% crystal violet for 15 min.

**Transwell Assay**

The T24 cells were collected and adjusted to 5 × 10^4^ cells/well. The cells were inoculated into the upper chamber of a Transwell plate, and the lower chamber was supplemented with 10% FBS medium. The plate was placed in the incubator for 24 h, washed twice with PBS, and fixed with methanol. Then, the cells were treated with 0.5% crystal violet staining solution, washed three times with PBS, and allowed to air dry. The number of migrated cells was recorded by photography under multiple high-magnification fields using a microscope and by counting the number of migrated cells.

**Peripheral Blood Mononuclear Cells (PBMC) isolation and culture**

Peripheral blood samples were collected from healthy adult donors who provided informed consent after being informed of the study objectives. Blood (4 mL per tube) was collected using EDTA anticoagulation tubes and processed within 2 hours. The Human Peripheral Blood Lymphocyte Separation Solution and Wash Buffer (TBDscience, China) were employed for isolation. A 15 mL centrifuge tube was prepared, and an equal volume of separation solution was added. The blood sample was gently layered onto the upper layer of the separation solution using a pipette, followed by centrifugation at 500 g for 25 minutes. After centrifugation, the blood separated into four layers. The second layer, a milky-white lymphocyte band, was carefully aspirated using a pipette and transferred to a new 15 mL centrifuge tube. This was then mixed with 10 mL of wash buffer and centrifuged again at 250 g for 10 minutes, discarding the supernatant. The cells were resuspended in wash buffer (5 mL) and centrifuged at 250 g for 10 minutes, repeating this step twice. Finally, the cells were resuspended in 0.5 mL of 10% FBS LymGro lymphocyte culture medium (Invitrogen, USA), counted, and seeded at a concentration of 1×10^6^ cells/mL into T25 flasks for cultivation.

**Co-culture Experiment**

25 ul/ml of ImmunoCult^TM^ Human CD3/CD28/CD2 T Cell Activator (10970, STEMCELL Technologies, Vancouver, Canada) and 10 ng/ml of rhIL-2 (202-1L-050, R&D Systems, USA) were added to activate T cells. Cell counting and media exchange with fresh lymphocyte culture medium were performed every 2-3 days to maintain cell viability. After 5-6 days, bladder cancer cell lines from each group were seeded at a concentration of 1×10^5^/ml, 1 ml/well in 24-well plates and allowed to adhere overnight. Then, 3×10^5^ PBMCs per well were added along with 100 ng/ml of anti-huamn CD3 (16-0037-81, Invitrogen, USA) for complete T cell activation. Simultaneously add 2 μl of Cell Activation Cocktail (phorbol 12-myristate 13-acetate/ionomycin/brefeldin A, 423303, BioLegend, San Diego, CA) to promote the secretion of intracellular cytokines and prevent their excretion from the extracellular environment. The two types of cells were co-cultured at 37°C in an atmosphere containing 5% CO_2_ for approximately 48 hours. Following incubation, supernatants from each well were aspirated using a pipette for flow cytometry analysis. Adherent cells were fixed with 4% paraformaldehyde solution for 30 minutes, washed twice with PBS, stained with 0.2% crystal violet solution for 30 minutes, rinsed with water to remove excess dye, air-dried, and then photographed under a microscope for documentation.

**Chemotaxis Assay**

Bladder cancer cell lines were seeded at a density of 5×10^4^ cells per well in a 24-well plate and cultured for 36 hours in RPMI-1640 medium containing 10% FBS. Subsequently, a chemotaxis assay was performed using a Transwell chamber with a 3 μm pore size. In the upper chamber, 5×10^5^/ml, 200 μl of activated CD8^+^ T cells were added, while in the lower chamber, 600 μl of the supernatant from the different bladder cancer cell lines, after 36 hours of culture, were placed. The chambers were incubated at 37°C in an atmosphere of 5% CO_2_ for 8 hours. Afterward, the cells in the lower chamber were collected and counted.

**Flow cytometry**

(1) The co-cultured supernatant was centrifuged at 1500 rpm for 5 minutes, resuspended in PBS for cell counting, and then each group of cells with the same count (1×10^6^ cells) was placed in EP tubes, centrifuged again at 2000 rpm, 4°C for 5 minutes;

(2) Resuspend in 100 μl PBS, add 1ul Zombie Aqua Fixable Viability (biolegend, China) to each tube, tapped gently, incubate in the dark at 4°C 20 minutes, add 1ml of 2% FBS PBS to stop the reaction, centrifuge at 2000 rpm, 4°C 5 minutes, discard the supernatant, repeat once.

(3) Resuspend in 100 μl of 2% FBS-PBS, add 1ul of human Fc receptor blocking antibody (biolegend, China) to each tube to avoid non-specific staining, gently mix, incubate at 4°C in the dark for 15 minutes, centrifuge at 2000rpm, 4°C for 5 minutes, discard the supernatant;

(4) Prepare antibody in 2% FBS PBS (100 μl per tube), incubate at 4°C in the dark for 30 minutes, centrifuge at 2000rpm, 4°C for 5 minutes, wash once with 1ml of 2% FBS PBS, centrifuge, discard the supernatant;

(5) Use the membrane permeabilization reagent kit (BD, China) to permeabilize the cell membrane and nuclear membrane, add 250ul of fixation/permeabilization solution to each tube, incubate at 4°C for 20 minutes, then wash twice with 1×BD perm/washTM buffer;

(6) Resuspend in 100ul of 1×BD perm/washTM buffer, add intracellular antibodies, incubate at 4°C in the dark for 50 minutes, wash twice with PBS, discard the supernatant, resuspend in 500 μl of PBS for analysis.

**TissueFAXS Comprehensive Quantitative Analysis**

We stained for ACSM6 (PA5-60768, Invitrogen, Carlsbad, USA), and CD8 (66868-1-Ig, Proteintech, Deansgate, USA) using specific primary and secondary antibodies, while staining tumor cells with CK19 (10712-1-AP, Proteintech, Deansgate, USA), fibroblasts with PDGFR-alpha (ab203491, Abcam, Combridge, USA) and cell nuclei with DAPI (ab104139, Abcam, Combridge, USA) by Biomark Biology company (Beijing, China). To evaluate the relationship between ACSM6^+^, CK19^+^, and CD8^+^ cells, we performed quantitative analysis of co-expression in multiplex immunofluorescence staining using TissueFAXS Panoramic software (TissueGnostics, Austria).
